# Progress in State Medicine

**Published:** 1900-01-06

**Authors:** 


### Progress in State Medicine.

**Meat Inspection.**—Thompson[1] formulates the following scheme of requirements for thorough meat inspection. Public abattoirs should be established in all sufficiently populated areas, their initial cost being such as to render them self-supporting. The Government may compel the local authority to make such provision, and shall lend the necessary capital. The inspectors employed must be independent of local influences. All meat sold within the area must bear an official stamp. A class of educated inspectors must be created. The Glebe Island abattoir at Sydney, after many years of failure, was taken over by the Board of Health; and during the past year, after spending £800 on repairs, showed a profit of £2,500.

The abolition of private slaughter-houses has been advocated by the Royal Commission on Tuberculosis, the Public Health Committee of the London County Council, and by the medical officer of the Local Government Board, the chief obstacle to the reform being, as Manly[2] states, the condition of public opinion on this question. Liverpool, with a population of 668,000, has only 30 private slaughter-houses, and these are visited by five inspectors. Few English towns are as well equipped as this however, and all compare badly with Continental cities such as Berlin and Leipzig, where no private slaughtering is permitted, all the work being done in public halls. All meat brought into these towns is taken to the central office for inspection, and may be subjected to microscopical examination. All meat passed as sound is stamped in several places with the official stamp, and bad meat destroyed by the authorities. The number of stamp marks is proportional to the size of the carcass. Thus a carcass of beef has 12 stamps, a carcass of mutton eight stamps. Food of an inferior quality is sold by special dealers, at prices fixed by the authorities, in a special building known as the Freibank. At such institutions, too, is sold the flesh of animals slightly diseased, which has been thoroughly boiled on the premises. In Germany compensation is paid for closing private slaughter-houses, the amount being fixed by arbitration, and being dependent on the nature of the trade and buildings.

The effect which the establishment of public abattoirs would have on the spread of tuberculosis in London is reviewed by Bond.[3] He refers to Martin's statement that a small dose of tuberculous material may infect the internal organs without producing a lesion of the mucous membrane by which it is absorbed as explaining cases of tuberculous adenitis, peritonitis, &c. Probably 40 per cent. of cows and 15 per cent. of swine are tuberculous. Public abattoirs would be a benefit to the stockowner, because he would gradually eliminate tuberculous strains from his stock; all butchers could then slaughter home-fed animals; there would be an increased demand for home-fed meat, which would be guaranteed sound. The butcher would benefit also from the increased demand, the monopoly of private slaughter-houses would be abolished; meat in hot weather would be stored in the public cooling chambers, so saving the cost of ice. The public would benefit in having more wholesome meat; the nuisance of slaughter-houses near private dwellings would be abolished; putrefiable matters would be excluded from the sewers; blood and offal now lost would be utilised, a profit would accrue to the community; much cruelty to the beasts would be abolished. In Holborn one-third of the meat "seized" has been for tuberculosis; the total weight per annum in this district is 16,000 stone.

At the conference of veterinary inspectors Hunting[4] advocated the wider employment of veterinary experts. He instanced the services rendered by the Veterinary Department in India, South Africa, and in the rinderpest outbreak of 1865. Only a few local authorities have appointed veterinary advisers, although the diseases common to man and animals are so many and so varied. He instanced rabies, anthrax, tuberculosis, trichinosis, ringworm. In the inspection of all carcases, hides, hair, and wool, local authorities need veterinary assistance. Injustice to traders may result from ill-trained officers, and the Public Health (Scotland) Act has recognised the veterinary surgeon as an essential officer of the hygienic service. Every local authority has a veterinary inspector under the Diseases of Animals Act; what is required is that his services shall also be obtainable under the Public Health Act.

There has always been difficulty in defining the exact duties of meat inspectors, and the position they should take under the health authorities. No better model could be taken than the pamphlet on the duties of a meat inspector recently issued by the authorities of St. Mary's parish, Islington.[5]

**Epidemic Diarrhœa.**—Newsholme[6] states that epidemic diarrhœa is responsible for 4·8 per cent. of deaths from all causes. The highest mortality occurs in the third quarter of the year, but this seasonal alteration of severity is very different in various towns. The essential cause is undoubtedly a micro-organism, probably the B. enteritidis sporogenes. It is essentially a disease of urban life, but density of population does not bear any direct proportion to diarrhœal mortality. The artisan class suffers most heavily, and the employment of married women is a factor. There is less fatal diarrhœa in towns where water-carriage system of excrement disposal is adopted, and where house refuse removal and street scavenging is efficient. Whilst loose soils are more dangerous than rocky soils, this danger may be counteracted by good drainage and paving. A high temperature and scanty rainfall were conducive to an outbreak. Epidemic diarrhœa was essentially a filth disease, dependent on polluted surface soil. It is largely spread by dust. Newsholme's acceptance of the causal relationship existing between the B. enteritidis sporogenes and epidemic diarrhœa is of interest in connexion with Klein's[7] work on this organism. Klein found the organism also in cases of ordinary diarrhœa and cholera nostras, but not in the stools of healthy persons. It is present in the sewage of our large towns, even after precipitation, sedimentation, and filtration. It readily lives in river water. It may exist for prolonged periods on horse dung, in garden earth, and in dust. The B. enteritidis sporogenes is allied to the bacillus of malignant œdema, but its power of rapidly forming gas in milk cultures, and of separating the milk into whey and casein is characteristic of the former organism. The organism is rapidly fatal to guinea-pigs. In the well-known outbreak at St. Bartholomew's Hospital the cases due to infection by this bacillus were characterised by profuse, slimy, and sometimes bloody evacuations, with colic. No case ended fatally.

[1] Public Health, Oct.. 1899. [2] Brit. Med. Jour., Sept. 2, 1899. [3] Jour. Sanit. Inst., July, 1899. [4] Jour. Sanit. Inst., Oct., 1899. [5] Sanit. Record, Oct. 20, 1899. [6] Brit. Med. Jour., Nov. 25, 1899. [7] Centrab. für Bakter. xxiii. 13.

